# A multi-criteria decision analysis of management alternatives for anaerobically digested kraft pulp mill sludge

**DOI:** 10.1371/journal.pone.0188732

**Published:** 2018-01-03

**Authors:** Martijn Eikelboom, Alice do Carmo Precci Lopes, Claudio Mudadu Silva, Fábio de Ávila Rodrigues, Antônio José Vinha Zanuncio, José Cola Zanuncio

**Affiliations:** 1 Departamento de Engenharia Florestal, Universidade Federal de Viçosa, Viçosa, Minas Gerais, Brasil; 2 Departmento de Engenharia Civil, Universidade Federal de Viçosa, Viçosa, Minas Gerais, Brasil; 3 Departamento de Química, Universidade Federal de Viçosa, Viçosa, Minas Gerais, Brasil; 4 Departamento de Engenharia Florestal, Universidade Estadual do Centro-oeste, Irati, Paraná, Brasil; 5 Departamento de Entomologia, Universidade Federal de Viçosa, Viçosa, Minas Gerais, Brasil; Tallinn University of Technology, ESTONIA

## Abstract

The Multi-Criteria Decision Analysis (MCDA) procedure was used to compare waste management options for kraft pulp mill sludge following its anaerobic digestion. Anaerobic digestion of sludge is advantageous because it produces biogas that may be used to generate electricity, heat and biofuels. However, adequate management of the digested sludge is essential. Landfill disposal is a non-sustainable waste management alternative. Kraft pulp mill digested sludge applied to land may pose risks to the environment and public health if the sludge has not been properly treated. This study is aimed to compare several recycling alternatives for anaerobically digested sludge from kraft pulp mills: land application, landfill disposal, composting, incineration, pyrolysis/gasification, and biofuel production by algae. The MCDA procedure considered nine criteria into three domains to compare digested sludge recycling alternatives in a kraft pulp mill: environmental (CO_2_ emission, exposure to pathogens, risk of pollution, material and energy recovery), economic (overall costs, value of products) and technical (maintenance and operation, feasibility of implementation). The most suitable management options for digested sludge from kraft pulp mills were found to be composting and incineration (when the latter was coupled with recycling ash to the cement industry). Landfill disposal was the worst option, presenting low performance in feasibility of implementation, risk of pollution, material and energy recovery.

## Introduction

Brazil holds one of the world’s major shares in the pulp and paper export market with 17.2 million tons of pulp produced annually [1]. Kraft pulping, the most common pulp producing process in Brazil, demands approximately 30 m^3^ of water per ton of pulp produced. This process generates effluent with high organic content that cannot be discharged without treatment [2; 3]. A typical kraft pulp mill effluent treatment plant produces about 40 kg of primary sludge and 15 kg of secondary sludge per ton of dry pulp. In countries like Brazil, China and USA, both sludges are normally disposed into landfills, but could be used to produce biogas [2,3].

The waste stream that is digested to produce biogas produces a waste stream that is dewatered, resulting in a liquid and a solid fraction (biosolids). The liquid fraction is typically used to product biofuel and fertilizer, while the solid fraction is typically disposed of by land application, incineration or landfilling (S1 Fig) [3–5].

The objective of this study was to investigate, compare and select, the most suitable options for managing anaerobically digested primary and secondary sludges from kraft pulp mills, using the Multi-Criteria Decision Analyzing (MCDA) procedure.

## Material and methods

Six alternatives for recycling pulp mill digested sludge were examined, based on the most common management alternatives adopted in the USA, China and Brazil: land application, landfill disposal, composting, incineration, pyrolysis/gasification, and biofuel production by algae [2,6,7,8]. The study data were obtained from published literature, and the recycling alternatives were compared using MCDA, a procedure widely accepted in solid waste management studies [9,10]. The method compares various alternatives and considers the opinion of stakeholders.

To evaluate the recycling alternatives, were considered environmental, economic and technical aspects, in order to find the best alternatives for sludge treatment. The environmental criteria considered possible environmental damage from sludge treatment, including the following decision criteria: CO_2_ emission; exposure to pathogens; pollution risks; material recovery; and energy recovery. The CO_2_ emissions were calculated using previously developed equations for landfill disposal, land application, and composting [11]. Exposure to pathogens, risk of pollution, and material and energy recovery were based on the data in published literature and previous research [12–18].

The overall costs criteria (costs for operation, maintenance, transportation, labor, energy demand and, in some cases, quality control or soil testing) and product value were selected based on the economic criteria. The costs for all alternatives except algae production, were selected based on data from Stamatelatou and Tsagarakis [16]. The product value was calculated using the average market value in the USA of the product recovered [13–16].

Technical criteria were selected to ensure the feasibility of each recycling option for the kraft pulp mill industry. The criteria for maintenance and operation, and for the feasibility of implementing an option in kraft pulp mills, were chosen for this purpose. Maintenance and operation refers to the recycling process and to the complexity of the alternatives proposed. Implementation feasibility for kraft pulp mills refers to the viability of adapting available management options to a typical kraft pulp mill.

The criteria were assigned different weight factors (WF1, WF2 or WF3) to denote the perceived importance of the criteria. The feasibility of implementing a digested sludge alternative in kraft pulp mills (WF3) was designated as the most important criterion, because it integrated the feasibility and adaptability of the technology to current industry practices. The overall costs, product values, and maintenance and operation criteria were each assigned a weight factor of two (WF2), according to their economic attractiveness and feasibility importance. The weight factor of one (WF1) was assigned to criteria for CO_2_ emission, exposure to pathogens, pollution risks, material recovery, and energy recovery.

The options were ranked from one to six, i.e., from the worst (one) to the best (six), based on the literature data and calculations. The calculated sum of each recycling alternative was determined using the weight assigned per criterion. The higher the sum, the better was the recycling alternative.

Delphi questionnaires to MCDA evaluations were prepared [17] and an electronic survey was sent to a number of academic and non-academic experts in the field from the environmental engineering to support the analysis. The participants were asked to rank the alternatives described per criterion in a preference order. From them, eight volunteered to contribute with the survey.

An anaerobic digestion model [18] was used with the software Aspen Plus^®^, System number: SYS917070, to estimate the digested sludge generation by the anaerobic digestion of kraft pulp mill primary and secondary sludges, and the mixture between them (2.5:1 ratio, in total solids basis). The kraft pulp mill in study is located in the state of Minas Gerais, Brazil. Water [19], proteins [20], lipids [20] and ash content [19] were characterized in the sludge. The cellulose and hemicellulose contents of primary [21] and secondary [22] sludge were based on data from published literature that examined sludge from kraft pulp mills [8,23,24]. The liquid and solid fractions of the kraft pulp mill digested sludge were measure from the water fraction provided by the model in Aspen Plus^®^.

## Results

The characteristics of primary and secondary kraft pulp sludges, and their mixture, are presented in S1 Table. Both have high concentrations of fibers (cellulose and hemicellulose) that are potential substrates for bacteria in the anaerobic digestion process. Nevertheless, their protein content (i.e., nitrogen concentration) is low. The lack of nitrogen impairs biogas production, because it is an essential element for bacterial growth.

The characteristics of the digested sludge predicted by the model simulation highlights the efficiency of anaerobic digestion of secondary pulp mill sludge in comparison with digestion of primary sludge. More residual solids remained after digestion of the primary sludge compared to secondary sludge, which means that there is unused potential for biogas production from primary sludge due to the lack of nitrogen in this type of sludge. This fact is verified in the production of methane, where secondary sludge presents 72% higher production (S2 Table). The production of methane can be an interesting alternative for energy use [25,26].

Using MCDA to evaluate the combined environmental, economic and technical domains of alternatives, the options were ranked from best to worst as follows: composting (1); incineration (2); land application (3); pyrolysis/gasification (4); algae production (5) and landfill disposal (6) (S3 Table).

## Discussion

### CO_2_ emission

For primary sludge, the CO_2_ emissions from landfill disposal, land application and composting were estimated as 0.23, 0.60, 0.16 kg CO_2_/kg digested sludge, respectively. Cement production was the worst alternative in terms of CO_2_ emission [27]. The CO_2_ emission of crop wastes pyrolysis was lower compared to fossil fuels [28]. For gasification, the digested sludge is converted into CO, H_2_ and CO_2_ at a high temperature and the mixture of these gasses can be combusted to reduce the CO_2_ emission [29]. The CO_2_ emission reported in literature was 190 kg CO_2_/MWh from gasification of walnut waste [30]. The CO_2_ emissions from thermal recycling processes and algae production were not calculated due to lack of data. However, it is expected that algae production would emit less CO_2_ than would pyrolysis/gasification and incineration, because algae capture CO_2_.

### Exposure to pathogens

For landfill disposal, the risk of exposure to pathogens is low if impermeable linings protected by sand layers are applied to prevent leaching of contaminants to groundwater [31]. Land application, without pre-treatment, showed a significantly high risk of pathogen exposure. Therefore, sludge and digested sludge have to meet quality standards regarding heavy metals, pathogens and vectors [32]. The high temperatures for thermal recycling alternatives should inactivate pathogens [33]. Nevertheless, pathogens inactivation also happens at relatively low temperatures (50°C) in a composting pile [34]. For algae production, the exposure to pathogens could cause occupational health or environmental problems [35].

### Risk of pollution

For landfill disposal, harmful contaminants can leach through the soil, polluting groundwater and surface water. In addition, nutrients (N, P, K, Ca and Mg) at high concentrations can leach to groundwater [36,37]. The heavy metal content in digested kraft pulp sludge does not exceed legal limits [38], but potentially toxic elements in the kraft pulp mill digested sludge are a risk in land application [3]. Heavy metals accumulate in agricultural soil and their persistence in topsoil causes problems in the food chain [39]. Composting decreases the organic matter content and dissolved organic carbon, resulting in high heavy metal concentration in the final compost [40]. Cement production from digested sludge oxidizes organic pollutants and immobilizes heavy metals [33]. For pyrolysis/gasification, digested sludge is first dried, pressed to pellets and then combusted. In the combustion, organic pollutants are oxidized, but heavy metals present in the feedstock will remain in the ash [41]. Algae-bacterial systems can remove organic pollutants, nutrients and heavy metals from wastewater streams [42]. However, well-mixed photobioreactors with algal biomass recirculation can protect algae from the toxicity of the liquid fraction.

### Material recovery

The disposal of sludge in a landfill is a waste of recyclable material that has both fertilizer and calorific value [43]. The land application and composting options allow the use of digested sludge in agricultural production as a low-cost soil amendment, but it is now becoming restricted due to the risk of pollution [3]. Incineration produces energy and ash from digested sludge [44], and dried sludge can be used to produce cement [45]. The heating value of the sludge is lower than the raw sludge due to decreased organic content after digestion, but incineration is still feasible [5]. Concerning pyrolysis, the kraft pulp mill digested sludge can be converted into bio-oil, pyrolysis gas and biochar. Bio-oil can replace crude oil, while pyrolysis gas can be used to produce energy, and biochar is a good soil conditioner [5]. The gasification process produces gas that can be used to produce electricity [46]. Algae production has potential applications, including biological CO_2_ sequestration and wastewater treatment [47], but its most interesting application is for biodiesel production [48].

### Energy recovery

Landfill disposal, land application and composting of sludge do not enable energy recovery. Raw sludge from wastewater treatment plants can be digested and incinerated. Houdková et al. [49] found the calorific value of digested sludge was only 2.1 MJ/kg. In a study conducted by Cao and Pawlowski [50], primary and secondary sewage sludges were digested and pyrolyzed, producing 0.102 ton bio-oil and 0.207 ton bio-char per ton of primary sludge, and 0.192 ton bio-oil and 0.407 ton bio-char per ton of secondary sludge. Although that study [50] was conducted using sludge from municipal wastewater treatment, it gives an insight in the energy production potential from kraft pulp mill digested sludges. Gasification of sludge was found to produce 8.197 MJ/kg sludge, which was a lower energy value than that of other feedstocks such as coal, vegetable oils, straw, wood and plants [51]. Biofuel production from algae grown using digested kraft pulp mill sludge as a substrate has not been reported.

The high moisture contents of both raw and digested sludges impair energy recovery through incineration and pyrolysis/gasification processes, but not through anaerobic digestion, which is efficient at relatively high moisture content.

### Overall costs

Overall costs of each alternative sludge management option were described in US dollars (US$) per ton of dry matter (S4 Table).

Overall costs for large-scale algae production from sludge have been poorly studied; however, these costs were estimated to be high due to maintenance and operation costs. Dewatering the digested sludge might increase the costs associated with incineration and pyrolysis/gasification due to the expected high moisture content of the kraft pulp digested sludge.

### Value of products

The revenue from digested sludge used for land application needs to be better studied; in a 1995 study, a revenue value of US$ 34–36 per ton was found [14]. The inflation from 1995 to 2016 changes this value to US$ 53–100 per ton of digested sludge. However, this was determined for treated sludge that was free of pathogens, heavy metals and odor; comparable data are scarce about the value of composted sludge. One ton of sludge dry solids (DS) were converted to 0.81 MWh through incineration [49]. One MWh of biomass or coal was valued in terms of the Brazilian real (R$) at R$ 251 [15]; therefore, the value of one ton of sludge DS is valued at R$ 203.31 (US$ 58.18). The ash value of sludge DS was estimated to be US$ 200 per ton [14]. One ton of digested sludge on a DS basis produced 0.17 ton of ash [49]. Therefore, the value of one ton of DS was set at US$ 34. Incineration of one ton of sludge DS is worth US$ 91.83.

Pyrolysis of one ton of digested primary sludge (DS basis) resulted in 0.102 ton bio-oil and 0.207 ton bio-char. The selling price for bio-oil and bio-char are US$ 0.66/L and US$ 0.4/kg, respectively [52]. The value of one ton digested primary sludge is US$ 80.84 for bio-oil, and US$ 82.80 for bio-char considering the density of the bio-oil to be 1.2 kg/L, resulting in a total value of US$ 163.64 per ton of digested primary sludge. One ton of digested secondary sludge (DS basis) produced revenue of US$ 314.96 [52]. Energy production from gasification was estimated to be 8,197 MJ per ton sludge, i.e., 2.277 MWh per ton sludge (DS) [51]. The Brazilian value of one MWh (R$ 251) [53] yields a revenue of R$ 571.53 (US$ 163.56) per ton dry sludge.

For algae production, 4,558.71 m^3^ of wastewater is needed for 1 m^3^ of biodiesel, and 1 m^3^ of biodiesel results in revenue of US$ 636.65. Therefore, 1 m^3^ of digested sludge (liquid fraction) is valued at US$ 7.16 [16].

### Maintenance and operation

*In-situ* composting is the preferred alternative regarding the maintenance and operation criterion. Neither land application nor landfill disposal is complicated, but each requires more maintenance in terms of professionals and quality control than does composting. Application of the digested sludge on land requires managers to minimize odor potential, pathogens and other harmful constituents in sludge to acceptable levels and frequently monitor possible environmental impacts using soil and groundwater analyses [54]. The kraft pulp mill digested sludge is too wet and needs to be dewatered [55]. The dewatering method needs to be further studied for kraft pulp mill waste because the anaerobic digestion process changes the capillary structure of the digested sludge, i.e., digestion alters the binding of water inside crevices and interstitial spaces that exist on and between particles and organisms [56].

Thermal treatment alternatives and algae production are more complex to operate than other alternatives. The kraft pulp mill digested sludge needs to be dewatered prior incineration. The dewatering requirement constitutes a major challenge because kraft pulp mill digested sludge has high moisture content. Gaseous emissions require air pollution control equipment. A major advantage of the thermal treatment alternative is to incinerate the kraft pulp mill in a biomass boiler. The bottom ash, a solid residue after incineration, can be used in cement production.

The relative complexity of pyrolysis processing equipment is the major disadvantage of this process. Pyrolysis involves a complex series of chemical reactions to decompose organic materials and produce oils, gases and char [57].

The major challenge of algae production is to implement an integrated system that combines large-scale production and algae harvesting to produce biofuels. Further investigation and development of large-scale production and harvesting methods for biofuels are necessary [58].

### Feasibility of implementing kraft pulp mill digested sludge

Landfilling of kraft pulp mill digested sludge is easily implemented; however, this alternative is outdated and has environmental risks, and does not accrue economic profits or facilitate any material or energy recovery. Land application of kraft pulp mill digested sludge is feasible to implement, but the possible pathogen contamination and heavy metal content need to be studied. Heavy metal content of raw kraft pulp sludge is low [38]. Composting allows reactors (i.e., compost piles) to be placed and operated on-site at a kraft pulp mill, if area is available. Incineration (which can take place in the biomass boiler of a kraft pulp mill) combined with ash utilization (in the cement industry) are promising solutions for managing kraft mill sludge. Pyrolysis and gasification of the digested sludge, when compared to incineration, have the disadvantage of being difficult to implement on-site at a kraft pulp mill [59]. In addition, these alternatives require high-cost investments. Thermal treatment is also an alternative of questionable feasibility because of the high moisture content in the kraft pulp mill digested sludge. Algae production seems a promising alternative, but more research is needed to determine its feasibility for managing digested sludge from a kraft pulp mill, since this type of sludge lacks some essential constituents, such as nitrogen. An option for solving this problem would be to apply a thermal pre-treatment [60] or ultrasound treatment [61] to solubilize the sludge.

## Conclusions

- Composting is the most suitable alternative for recycling the anaerobically digested sludge from kraft pulp mills.
- Composting is safe and produces low-cost fertilizer for agriculture. There is no energy recovery, but the overall costs are low and the process is feasible to implement.
- The incineration alternative may be easy to implement at a kraft pulp mill biomass boiler, because it includes energy recovery, and the ash generated can be recycled into cement production. Nevertheless, the incineration process is more complex and has higher costs compared to composting.
- The only difference between the opinion survey and the research based on literature and calculations was the score determined for the land application alternative, which was considered by the survey participants to be a better alternative than incineration.
- This study gave an insight into the advantages and disadvantages of various alternatives for managing anaerobically digested kraft pulp mill sludge.

## Supporting information

S1 Fig Processes for treatment and recycling anaerobic digested sludges (adapted). Source: SHEETS et al., 2015.
(TIF)

S1 Table Characteristics of the investigated kraft pulp mill sludge. ADt: air dry ton of pulp. PS: Primary Sludge. SS: Secondary Sludge. Mixed sludge ratio of 2.5:1 dry mass basis.
(PDF)

S2 Table Primary, secondary and mixed digested sludges production. (PDF)

S3 Table Weight factor (W), landfill disposal (Landfill), land application (Land App.), Composting (Comp.), incineration (Inc.), pyrolysis/gasification (P.G.) and algae from ranking the alternatives for each criteria. Values in parentheses are from the survey study.
(PDF)

S4 Table Alternative costs for handling the kraft pulp mill digested sludge. (PDF)

S1 Minimal Dataset Results from the survey. (XLSX)
