# Supplementary figures and images for: A multi-criteria decision analysis of management alternatives for anaerobically digested kraft pulp mill sludge
Source: PLoS One. 2018 Jan 3;13(1):e0188732. doi: 10.1371/journal.pone.0188732 (PMC5751971; doi:10.1371/journal.pone.0188732)

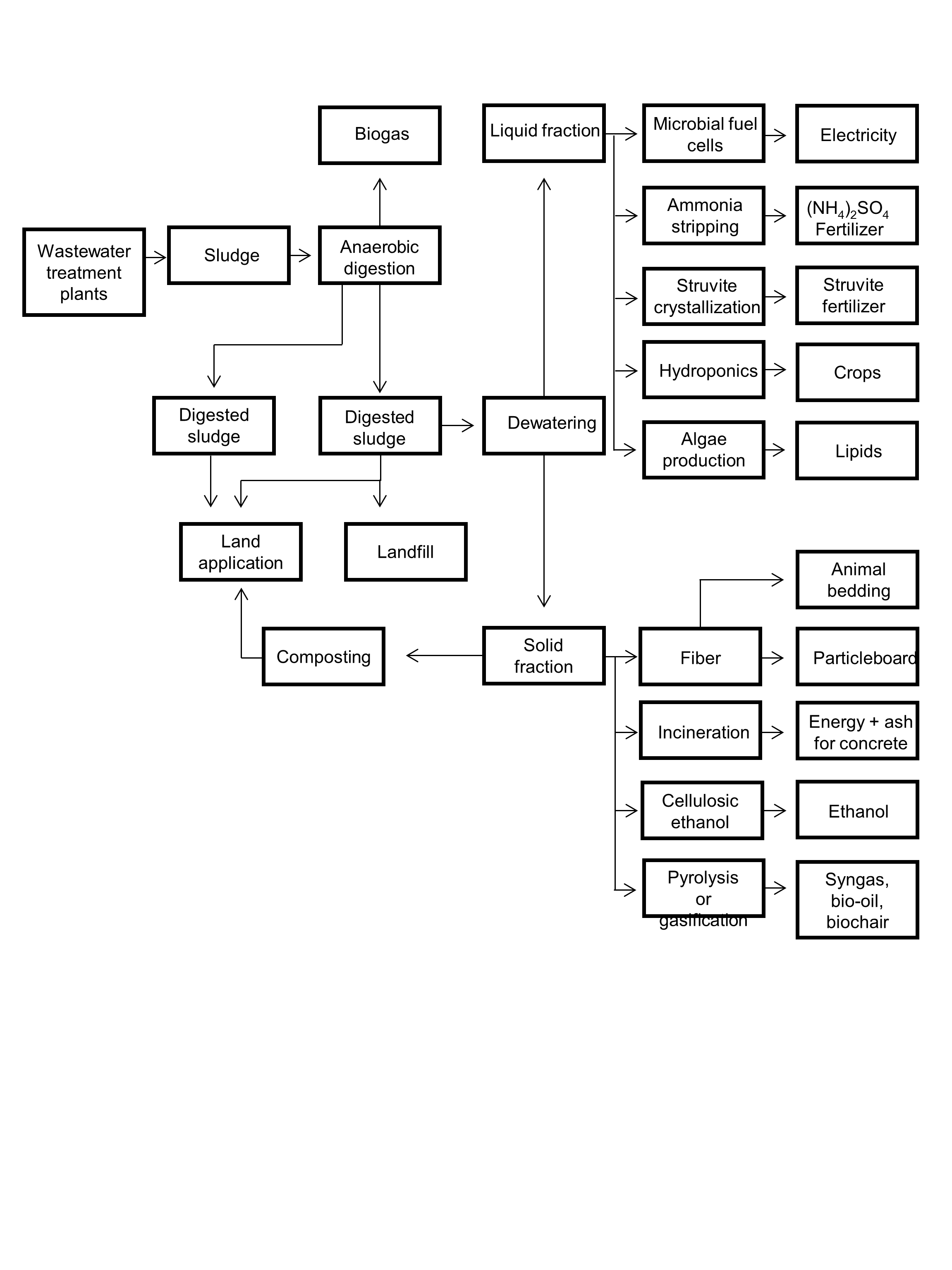

Supplement: S1 Fig — Source: SHEETS et al., 2015. (TIF) [file pone.0188732.s001.tif]
