# Supplementary material for: A multi-criteria decision analysis of management alternatives for anaerobically digested kraft pulp mill sludge
Source: PLoS One. 2018 Jan 3;13(1):e0188732. doi: 10.1371/journal.pone.0188732 (PMC5751971; doi:10.1371/journal.pone.0188732)
Supplement: S1 Table — ADt: air dry ton of pulp. PS: Primary Sludge. SS: Secondary Sludge. Mixed sludge ratio of 2.5:1 dry mass basis. (PDF) [file pone.0188732.s002.pdf]

Table 1: Characteristics of the investigated kraft pulp mill sludge

Parameters of sludge produced in the pulp mill	Primary	Secondary	Mixed sludge
Water (%)	61.52	88.63	77.12
Cellulose (%)	15.67	0.77	7.09
Hemicellulose (%)	3.07	1.44	2.13
Protein (%)	0.47	3.24	2.06
Lipids (%)	0.92	0.37	0.60
Ash (%)	18.35	5.56	11.04
Total (%)	100	100	100
Production			
Pulp production (ADt/d)		3,000	
Wastewater generation (m^3/d)		90,000	
Total sludge production (kg dry/ADt)		75	
Total sludge production (kg dry/d)		225,000	
Primary sludge production (kg wet/d)		409,304	
Secondary sludge production (kg wet/d)		593668	
Mixed sludge production (kg wet/d)		1002971	

ADt: air dry ton of pulp. PS: Primary Sludge. SS: Secondary Sludge. Mixed sludge ratio of 2.5:1 dry mass basis
