# Supplementary material for: A multi-criteria decision analysis of management alternatives for anaerobically digested kraft pulp mill sludge
Source: PLoS One. 2018 Jan 3;13(1):e0188732. doi: 10.1371/journal.pone.0188732 (PMC5751971; doi:10.1371/journal.pone.0188732)
Supplement: S2 Table — (PDF) [file pone.0188732.s003.pdf]

Table 2: Primary, secondary and mixed digested sludges production

	Primary	Secondary	Mixed
Total digested sludge production (kg/year.kg dry sludge)	866	2920	1538
Liquid production (kg/year.kg dry sludge)	555	2657	1227
Solid production (kg/year kg dry sludge)	311	263	311
CH_4 generation (kg/year.kg dry sludge)	70	121	88
Solids content (kg dry solids/kg weight solids)	0.386	0.09	0.202
Carbon content (kg C/kg dry solids)	0.460	0.360	0.430
