# Supplementary material for: A multi-criteria decision analysis of management alternatives for anaerobically digested kraft pulp mill sludge
Source: PLoS One. 2018 Jan 3;13(1):e0188732. doi: 10.1371/journal.pone.0188732 (PMC5751971; doi:10.1371/journal.pone.0188732)
Supplement: S3 Table — Values in parentheses are from the survey study. (PDF) [file pone.0188732.s004.pdf]

Table 3: Weight factor (W), landfill disposal (Landfill), land application (Land App.), Composting (Comp.), incineration (Inc.), pyrolysis/gasification (P.G.) and algae from ranking the alternatives for each criteria

Criteria	W	Landfill	Land App.	Comp.	Inc.	P.G.	Algae
Lowest CO_2 emission	1	$4^{(1.3)}$	$3^{(3.0)}$	$5^{(3.0)}$	$1^{(2.4)}$	$2^{(1.9)}$	$6^{(4.1)}$
Lowest exposure to pathogens	1	$4^{(1.6)}$	$2^{(1.2)}$	$5^{(2.6)}$	$6^{(3.2)}$	$6^{(3.8)}$	$3^{(3.4)}$
Lowest risk of pollution	1	$1^{(1.9)}$	$2^{(1.7)}$	$4^{(3.4)}$	$6^{(2.6)}$	$5^{(2.6)}$	$3^{(3.6)}$
Material recovery	1	$1^{(0.8)}$	$2^{(2.0)}$	$3^{(2.7)}$	$6^{(3.5)}$	$5^{(3.3)}$	$4^{(3.5)}$
Energy recovery	1	$3^{(1.6)}$	$3^{(1.7)}$	$3^{(2.4)}$	$4^{(3.0)}$	$6^{(3.8)}$	$5^{(3.3)}$
Lowest overall costs	2	$8^{(7.1)}$	$10^{(8.4)}$	$12^{(6.2)}$	$6^{(4.1)}$	$4^{(2.6)}$	$2^{(3.0)}$
Value of products	2	$2^{(1.5)}$	$4^{(4.9)}$	$6^{(5.4)}$	$10^{(6.8)}$	$12^{(6.8)}$	$8^{(6.2)}$
Lowest maintenance and operation	2	$8^{(6.2)}$	$10^{(7.3)}$	$12^{(6.2)}$	$6^{(4.5)}$	$2^{(3.4)}$	$4^{(3.9)}$
Feasibility of implementation to kraft pulp mill	3	$3^{(5.9)}$	$15^{(8.7)}$	$18^{(8.2)}$	$9^{(8.4)}$	$6^{(9.6)}$	$12^{(6.5)}$
Total		$34^{(28.0)}$	$51^{(38.9)}$	$68^{(40.2)}$	$54^{(38.5)}$	$48^{(37.6)}$	$47^{(37.4)}$

Values in parentheses are from the survey study.
