# Supplementary material for: A multi-criteria decision analysis of management alternatives for anaerobically digested kraft pulp mill sludge
Source: PLoS One. 2018 Jan 3;13(1):e0188732. doi: 10.1371/journal.pone.0188732 (PMC5751971; doi:10.1371/journal.pone.0188732)
Supplement: S4 Table — (PDF) [file pone.0188732.s005.pdf]

Table 4: Alternative costs for handling the kraft pulp mill digested sludge.

**Alternative**	**Costs (US$.t^{-1})**	**Considered costs**
Landfill disposal	309	labor, vehicle fuel, electricity, landfill tax and gate fees
Land application	126–280	labor and regulatory testing of soil
Composting	90–160	labor
Incineration	332–441	labor, transport to site and quality control
Pyrolysis/gasification	332–441	labor, transport to site and quality control
Algae production	no data	–
